# A dominant suppressor mutation sheds light on TGN sorting for exocytosis

**DOI:** 10.1093/plcell/koae285

**Published:** 2024-10-22

**Authors:** Leonard Blaschek

**Affiliations:** Assistant Features Editor, The Plant Cell, American Society of Plant Biologists; Department of Plant & Environmental Sciences, University of Copenhagen, 1871 Frederiksberg C, Denmark

Correct localization is fundamental to the function of any macromolecule in the cell—be it protein, polysaccharide, or other. The trans-Golgi network (TGN) is a key hub for a multitude of intersecting vesicular transport routes. There, cargo is sorted for anterograde transport to the plasma membrane or vacuole, or retrograde transport back to the *cis*-Golgi or endoplasmic reticulum. Transport to these destinations occurs via multiple routes, involving numerous adaptors, membrane-fusion mediators (SNAREs), and modifiers, whose interactions ensure that each cargo molecule reaches its intended destination. Among these, ECHIDNA (ECH) was identified as a crucial player in the Arabidopsis (*A. thaliana*) secretory pathway more than a decade ago (Gendre et al. 2011). In *ech* mutant plants, exocytosis, but not endocytosis or vacuolar transport, is impaired, leading to severe growth defects. However, the mechanism of ECH action in the secretory pathway has remained elusive.

In new work in *The Plant Cell*, Anirban Baral, Delphine Gendre, and colleagues (Baral et al. 2024) identify TYPHON1 and 2 (TPN1/2), previously uncharacterized TGN-localized proteins that modulate the secretory pathway together with ECH. Initially, the authors used chemical mutagenesis to perform a suppressor screen of the *ech* mutant, recovering a dominant allele that restored wild-type growth (Figure). The causal mutation was a G163E substitution in TYPHON1, a DUF300 putative solute transporter with several transmembrane domains. Accordingly, expressing transgenic, native promoter–driven G163E variants of TPN1 (TPN^G163E^) or its close paralog, TPN2, in the *ech* mutant background also suppressed the *ech* phenotype. Fluorescent co-localization of TPN1 with TGN markers Clathrin Light Chain (CLC) and Vesicle Transport v-SNARE 12 (VTI12) showed that, like ECH, TPN1 was localized to the TGN. Crucially, wild-type TPN1 lost this TGN co-localization in the *ech* mutant background, while TPN^G163E^ was able to remain in the TGN even without ECH. Losing ECH and either of TPN1 or TPN2 led to exacerbated growth defects, while the *ech tpn1 tpn2* triple mutant could not be obtained. Curiously, the *tpn1 tpn2* double mutant grew similarly to the wild type as long as ECH was functional.

**Figure.**
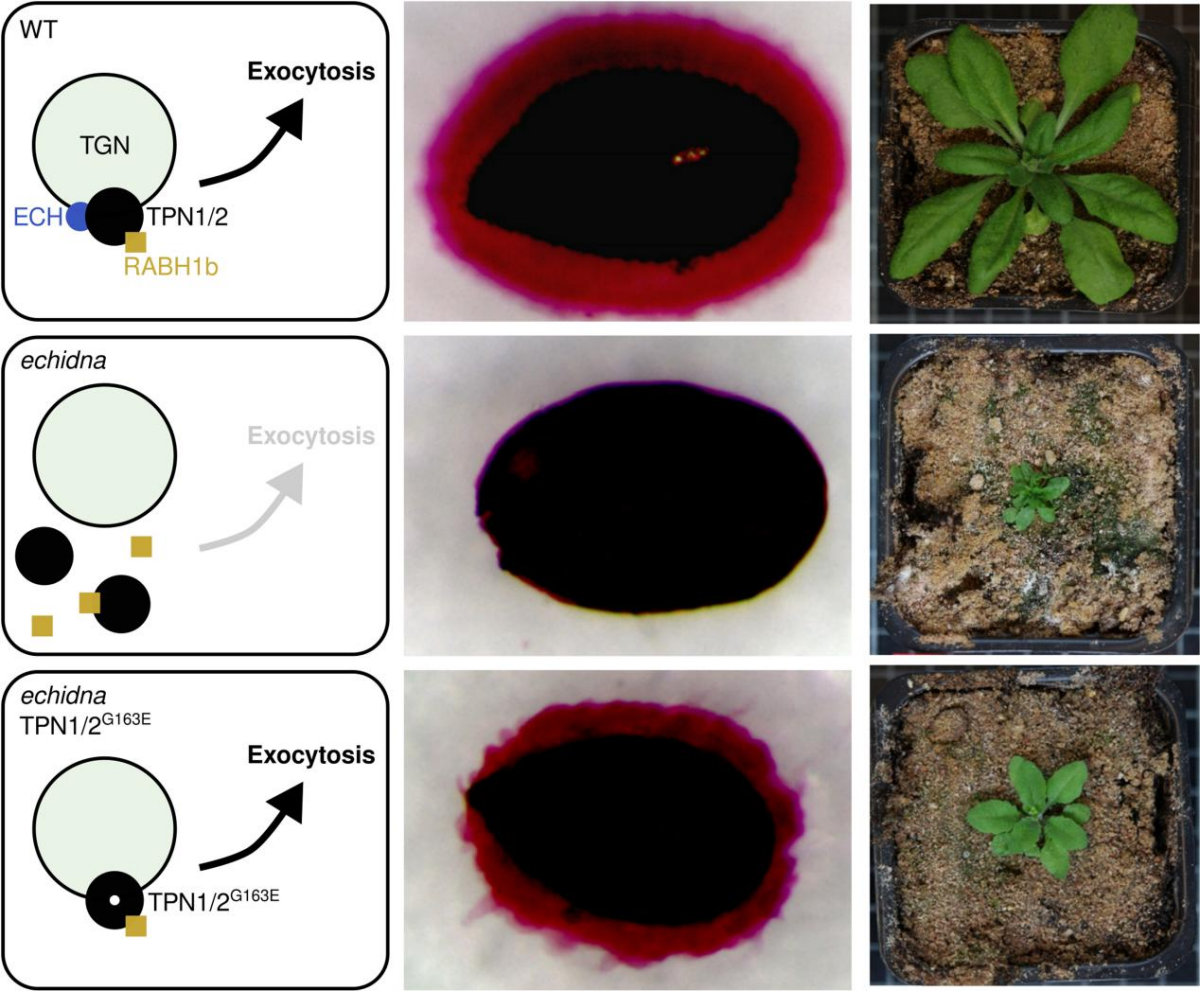
Model of TPN1/2 function. In the proposed model, TPN1/2 require ECH for TGN localization and efficient exocytosis. Loss of ECH drastically impedes exocytosis, leading to pleiotropic effects, including a drastic reduction in seed coat mucilage and plant growth. A point mutation in TPN1/2 leads to their ECH-independent TGN localization and partially restores exocytosis. Images adapted from Baral et al. (2024).

Both the *Caenorabdhitis elegans* ortholog of TPN1 and the Arabidopsis ECH-interactor YIP4 modulate the dynamics of RAB-GTPases (Gendre et al. 2013), suggesting a conserved involvement in ECH/TPN function. RABH1b, which had previously been shown to interact with YIP4, indeed showed mislocalization away from the TGN in the *ech* mutant, which could be rescued by TPN1^G163E^. Further, the *rabh1b* mutant phenotype appeared epistatic to both ECH and TPN1/2, with or without the G163E substitution, showing that the functions of ECH and TPN1 require RABH1b. Baral et al. (2024) propose that the TPN proteins, while not required for ECH function in the wild type, can compensate for the loss of ECH as long as they were retained in the TGN by a G163E mutation. Either ECH or TPN^G163E^ are sufficient for the function of RABH1b in the TGN, maintaining efficient transport through the secretory pathway.

How the TGN ensures that cargo reaches its destination and balances the endo- and exocytic flux through the endomembrane system remains perplexing (Yan et al. 2021). It has recently been shown that the TGN has several subdomains, which serve distinct transport routes and are characterized by specific adaptor and SNARE proteins. The TGN markers CLC and VTI12 used in the present study would likely associate with the clathrin-coated secretory zone and the vacuolar trafficking zone, respectively (Shimizu et al. 2021; Dünser et al. 2022). ECH, on the other hand, has previously been suggested to localize to uncoated secretory vesicles (Boutté et al. 2013), perhaps explaining the relatively limited co-localization of TPN1 with the above markers. Building on this work and establishing the precise functional interactions of ECH and TPNs—including TGN zone, adaptor proteins, SNAREs, and membrane-lipids—will likely bring us closer to a mechanistic understanding of these intriguing proteins and TGN sorting in general.

## Data Availability

Not applicable.

## Dive Curated Terms

The following phenotypic, genotypic, and functional terms are of significance to the work described in this paper:

ECH Gramene: AT1G09330
ECH Araport: AT1G09330
